# New vaccination approach using formalin-killed *Streptococcus pyogenes* vaccine on the liver of *Oreochromis niloticus* fingerlings

**DOI:** 10.1038/s41598-024-67198-0

**Published:** 2024-08-07

**Authors:** Sameh Nasr-Eldahan, Mohamed Attia Shreadah, Adham M. Maher, Tamer El-Sayed Ali, Asmaa Nabil-Adam

**Affiliations:** 1 Oceanography Department, Faculty of Science, Alexandria University, Alexandria, Egypt; 2 Marine Biotechnology and Natural Products Laboratory, National Institute of Oceanography & Fisheries, Cairo, Egypt; 3 Biochemistry Department, Faculty of Science, Alexandria University, Alexandria, Egypt

**Keywords:** Vaccine, Liver, *Streptococcus pyogenes*, *Nile tilapia*, Fingerlings, Microbiology, Zoology, Animal biotechnology, Vaccines

## Abstract

Newly synthesized vaccines prepared from formalin-killed bacteria *Streptococcus pyogenes* were investigated in the current study to evaluate the effectiveness of the newly synthesized vaccine as well as their safety by injected intraperitoneal. The study involved several steps 1st step is the preparation of the vaccine followed by the 2nd step: Evaluate the effectiveness and vaccine safety against pathogenic *S. pyogenes* through 4 different groups including control (Group I). Group II (Bacterial, infected group), Group III (Vaccine), and the Last group was the challenged group after the vaccination (Vacc + Bac). Different Immunological and biochemical parameters were measured in addition to hematological and histopathological examinations. For example, oxidative/antioxidants, inflammatory biomarkers, fragmentation and cell damage, and finally the histopathological study. The current study showed an increase in all oxidative, inflammatory, and cell damage (DNA fragmentation assays), additionally markedly elevation in histopathological cell damage in the infected group (Group II) compared with the control group. The vaccine and challenged after vaccination group (vaccine + Bacteria), showed great improvement in oxidative biomarkers (LPO) and an increase in antioxidants biomarkers (GSH, SOD, GST, DPPH, ABTS, GR and GPx), Also the inflammation and histopathological examination. The newly synthesized vaccine improved the resistance of *Oreochromis niloticus* and can be used as a preventive therapy agent for pathogenic bacteria *S. pyogenes.*

## Introduction

In recent decades, *Nile tilapia* production has expanded significantly. Production of *Nile tilapia* rose by 3.3% in 2020, breaking the 6 million tons barrier and making more than 130 countries grow it. The ability of *Nile tilapia* to withstand poor water quality with low levels of dissolved oxygen, a wide range of temperatures and salinities, and high levels of ammonia, as well as its excellent market value and potential to replace marine fish species, may be to blame for the increase in *Nile tilapia* production worldwide^1,2^. Egypt ranks first in *Nile tilapia* production, 77% of the total output. Despite several problems, including illnesses, Egypt has the largest aquaculture sector in Africa and is the third-largest producer of *Nile tilapia* globally^3,4^. In *Nile tilapia* farming, transmitting diseases and parasites is one of the most challenging aspects. Several types of disease defenses are present in fish; including both innate and adaptive. An adaptive immune response specific to a particular pathogen is a specific defense^5^. By nature, *Nile tilapia intestines* contain bacteria. It is still possible for bacteria to be contagious when the conditions for culture and environment are favorable. Among the most frequently infected bacteria are *streptococcus, pseudomonas, and vibrio,* a gram-positive, non-motile bacterium that is thought to be the leading cause of aquaculture problems. If grown in fluid media, they are non-motile, do not produce spores, and grow in pairs or chains. For example, *streptococcus*, it typically attacks red blood cells, generating either total clearance (hemolysis). Moreover, it is a form of bacteria with a fermentative metabolism that predominantly generates lactic acid without generating any toxic gases or catalase^6^. *Streptococcus* diseases are caused by *S. pyogenes*, which causes various abnormal symptoms^7^, including abnormal behavior: Swirling that causes fish to become disoriented, lethargic, and bend their bodies. Eye lesions: Fish infected with these bacteria often develop eye lesions such as endophthalmitis, exophthalmia, and eye hemorrhages. External bleeding can be seen most commonly around the mouth or at the base of the fins, as well as reddish pigmentation around the anus or on the genital papilla. Acute *Streptococcus* outbreaks are often accompanied by abdominal fluid. Ascites and anus protrusion are commonly seen together. One of the best methods for preventing and controlling viral diseases in fish is vaccination, which works by boosting the immune system^8^. Vaccines have been widely utilized in aquaculture as a defensive mechanism against various infections to prevent the host from infection^9^. In vaccines; pathogenic organisms are rendered non-pathogenic by various methods to produce antigens. Vaccines work by stimulating the immune system of animals to be more resistant to diseases caused by common pathogens in the future. Vaccine-stimulated antibody-producing cells (B lymphocytes) remain sensitized and ready to respond to the agent if it ever gains entry^10^. Vaccines are often manufactured by killing the infectious agent in formalin and using it as an antigen to stimulate the immune system. Aquaculture vaccines are mostly killed vaccines. In addition to being less expensive, easier to make, stable in storage, and free of virulence. Most of the time, these vaccines target the outer layer of microorganisms or inner sections without losing the ability to replicate in the host^11^. As a result, this study evaluated an inactivated *S. pyogenes* vaccine for *Nile tilapia* to control streptococcal disease outbreaks for the first time, which were indicated by hematological parameters, immune biomarkers, and DNA fragmentation assays. Furthermore, antioxidant enzymes can be used as immune enhancement detectors.

### The study outcome

Our study aims to investigate an alternative therapy against microbial infections (*S. pyogenes*), by increasing *Nile tilapia* fish’s natural immunity and reducing the pathogenicity of microbial infection using the formalin-killed bacteria vaccine technique.

## Materials and methods

### Fish and rearing management

Healthy, 80 pathogen-free *Nile tilapia* fingerlings (15 ± 5) obtained from the National Institute of Oceanography and Fisheries (NIOF), were used in the current study. *Nile tilapias* were reared in 1000 L quarantine tanks in aerated fresh water at Marine Biotechnology and Natural Products Laboratory Animal House. A commercial diet (30% protein) was fed to the fish, which accounted for 4% of their total weight. Daily water cleaning and monitoring were conducted to maintain optimal fish conditions during the experiment, pH (7.5–8), ammonia (0.0016 ppm), dissolved oxygen (7 mg/l), and water temperature (28.1 C) were adjusted. The 25% of water beside the aeration was exchanged daily. Fish are housed for 4 weeks before the experiment begins to monitor their health. Three fish are necropsied randomly from each treatment. To ensure liver samples are free of *S. pyogenes* bacteria or other pathogens, samples are collected for bacteriological testing. The study is reported in accordance with ARRIVE guidelines and was approved by the animal ethics committee of the research ethics committee (ERC) of the Molecular Biology Research and Studies (MBRSI), Assiut University, Assiut, Egypt. With Rec. Number IOR G0010947-SCI-21-34-A”.

### Bacterial growth

One Litre of deionized water was combined with 15 g of pancreatic digest of casein, 5 g of peptic digest of soybean meal, 5 g of sodium chloride, and 15 g of agar to generate the growth medium. The components were combined by stirring the flask using a magnetic stirrer. The aliquot was divided into ten 100 ml flasks and autoclaved for 15 min at 121 °C in each of them to sterilize them. The flasks were preserved by being kept in a freezer at 4 °C. Trypticase Soy Agar (TSA) can be made into blood agar media by adding 5% sheep blood. Finally, *S. pyogenes* from the American Type Culture Collection (ATCC) In Virginia, United States, was added to the media. And media culture study was done.

### Vaccine preparation

*Streptococcus pyogenes* strain used for this study was purchased with ATCC19615 according to the methods described by Klesius et al.^12^, Pretto-Giordano et al*.*^13^, and Bastardo et al.^14^. The Strain was cultured and fermented in tryptic soy broth at 30 C for 72 h using a freshly made culture from an ATCC sample. Ten percent formalin solution was added to the culture in TSB to inactivate it, and after 24 h at 4 °C, the final concentration was 3%. To insure that the bacterial cells are inactive form 0.1 ml of the formalin-treated cultured was streaked on sheep blood agar. After being centrifuged at 7000 g for 30 min. at 10 °C with the inactivated culture, the cell pellet was re-suspended in sterile normal saline. The cell pellet was once again re-suspended in TSB after a second round of centrifugation, resulting in a final concentration of 2.0 × 10^7^ colony forming units (CFU) per milliliter. This CFU mL was calculated from colony counts following culture on TSA and measured using a spectrophotometer at a wavelength of 540 nm.

### Vaccine safety assessment

Ten fish per group received 0.2 ml of adjuvant vaccine intraperitoneally (I.P) to examine the vaccines’ potential side effects in vivo. MS 222 (50 ppm) was used to anesthetize the fish before usage^15^. Throughout the first week following injection, we notice any harmful effects, such as behavioral abnormalities, lesions close to the injection site, and immediate mortality. Necropsies were done on fish four and 8 weeks after they had been shot to assess the long-term consequences^16^.

### Experimental design (Immunization and challenge)

Step (I) Immunization Treatment: Healthy *Nile tilapia* After Acclimation and confirmation that they are free of bacterial pathogens, 80 fish (15 ± 5) were distributed into two groups (control) unvaccinated group which was injected with saline (0.1 ml), and the vaccinated group. The *Nile tilapia* *was* anesthetized using (MS222). *Fish* were injected intraperitoneal (IP) with 0.1 ml of the vaccine (0.1 × 10^7^ CFU). The vaccine group was injected twice at 21 days and then booster injections were injected at 32 days. Step (II) Challenge Treatment: Before the challenge, all fish were starved for 24 h and anesthetized with MS222 (100 mg/L). The fish were IP injected with 0.1 mL of bacterial suspension with a concentration of (0.1 × 10^7^ CFU/fish). A total of 10 fish from each duplicate were intraperitoneally injected with 0.1 mL of *Streptococcus Pyrogen* (approx. 10^7^ CFU mL^−1^). The survival rates were measured daily over a period of 2 weeks days of the experimental challenge, and dead fish were collected. All groups had blood taken before, during, and after the experiment.

### Hematological parameters indices

The hematological parameters indices such NLR, PLR, MPV, and MCV were determined using a hematological analyzer for animals.

### Routine biochemical parameters

The total protein was determined according to the method described by Hiller et al.^17^, and the total lipid was determined according to the method described by Zollner and Krisch’s^18^ method. Additionally, the total glucose was determined according to the method of Richardson^19^, the total protein, total lipid and total glucose were determined according to previous methods using Bio-diagnostic kits.

### Non-specific immune parameters

#### Nitric oxide (NO)

NO was determined using the Greiss reagent and served as an indicator of No production according to Wang et al.^20^.

#### Myeloperoxidase (MPO)

Peroxidase activity with 3, 3′, 5, 5′-tetramethylbenzidine (TMB, Sigma) was determined according to Suzuki et al.^21^.

### Oxidative/antioxidant biomarkers

The oxidative stress lipid peroxidation (LPO) was determined by measuring malondialdehyde (MDA) levels based on the reaction of MDA with Thiobarbituric acid according to Kei^22^. Different antioxidant biomarkers were evaluated in the current study Catalase (CAT): the assay was performed according to Hadwan,^23^. Superoxide dismutase (SOD) was determined by the indirect method using Pyrogallol as described by Marklund & Marklund^24^. Total reduced glutathione (GSH) the assay was performed according to Ellman^25^. Glutathione –S-transferase (GST) was determined according to the methods modified by Habig et al*.*^26^. Total glutathione reductase (GR) activity was determined by the method of Carlberg and Mannervik^27^. Assay of Glutathione Peroxidase (GPX) Glutathione peroxidase was determined by the method of JT^28^.

### Diphenylamine assay of DNA fragmentation and Vitamin C

The Diphenylamine assays were described by Dische^29^ and modified by Burton^30^. The level of Vitamin C was determined by the method of Omaye et al.^31^.

### Histopathological examination

Liver tissue specimens were taken from all individual groups at the end of the experiential period according to the method described by Carleton et al*.*^32^.

### Statistical analysis

For statistical analysis and graphing, Graph Pad PRISM software was used. A one-way analysis of variance was performed along with a Tukey post hoc test to determine the significance of the results. Alpha = 0.05 and denoted as *P* < 0.05 (*), *P* < 0.01 (**), or *P* < 0.001(***).

### Ethical approval

The authors declare that they have conducted all applicable laws, guidelines, and regulations from the government.

### Consent to participate

Authors informed.

## Results

### Routine biochemical parameters

**Figure 1:**
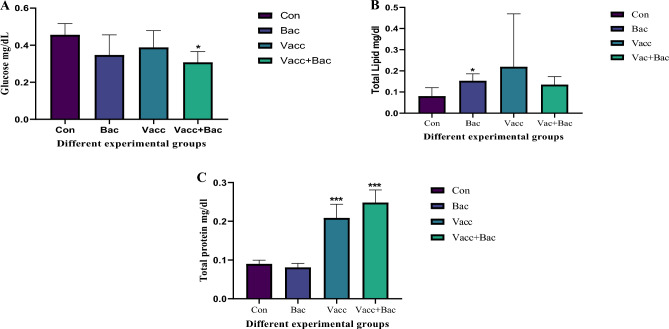
Different biochemical parameters (**A**) glucose (**B**) Total lipid and (**C**) total protein, Where the Con. The control (without treatment), Bac (the infected group with *S. pyogenes*), Vacc (the vaccinated group),Vacc + Bacc (Challenged group after vaccination).

The Total protein levels were evaluated and showed a nonsignificant decrease in the bacterial group compared with the control group (0.08083 ± 0.01048 Vs 0.08983 ± 0.009827, *P* = 0.6650), additionally, the Vacc and Vacc + Bac showed significantly increased compared with control (0.2087 ± 0.03509, *P* = 0.0006***& 0.2482 ± 0.03260, *P* = 0.0006***). Furthermore, the Vacc and Vacc + Bac showed significant increases compared with the bacterial group (0.2087 ± 0.03509, *P* = 0.0037** & 0.2482 ± 0.03260, *P* = 0.0003*** Vs 0.08083 ± 0.01048) Fig. 1C. The Total Lipid profile showed a significant increase in the Bacterial group compared with the control (0.1527 ± 0.03323 Vs 0.08035 ± 0.04015, *P* = 0.0018**) while the Vacc and Vacc + Bac showed a non-significant increase compared with the control (0.2190 ± 0.2506, *P* = 0.5387 & 0.1346 ± 0.03843, *P* = 0.1499 Vs 0.08035 ± 0.04015). Also, the Vacc and Vacc + Bac showed non-significant increases compared with the bacterial group (0.2190 ± 0.2506*, P* = 0.9039 & 0.1346 ± 0.03843,* P* = 0.8456 Vs 0.1527 ± 0.03323). The Glucose levels of Bacteria and vaccines showed non-significantly decreased compared to control groups (0.3468 ± 0.1088, *P* = 0.2646, 0.3880 ± 0.09097, *P* = 0.1713 Vs 0.4555 ± 0.06098). In contrast to Vacc + Bacteria showed significant decreases comparing with control (0.3074 ± 0.05832 Vs 0.4555 ± 0.06098, *P* = 0.008***). Also, the vaccine and vaccine Bacteria showed nonsignificant increases and decreases respectively compared with control (0.3880 ± 0.09097 & 0.3074 ± 0.05832 Vs 0.4555 ± 0.06098) (Fig. 1A).

### The antioxidant and oxidative stress

**Figure 2:**
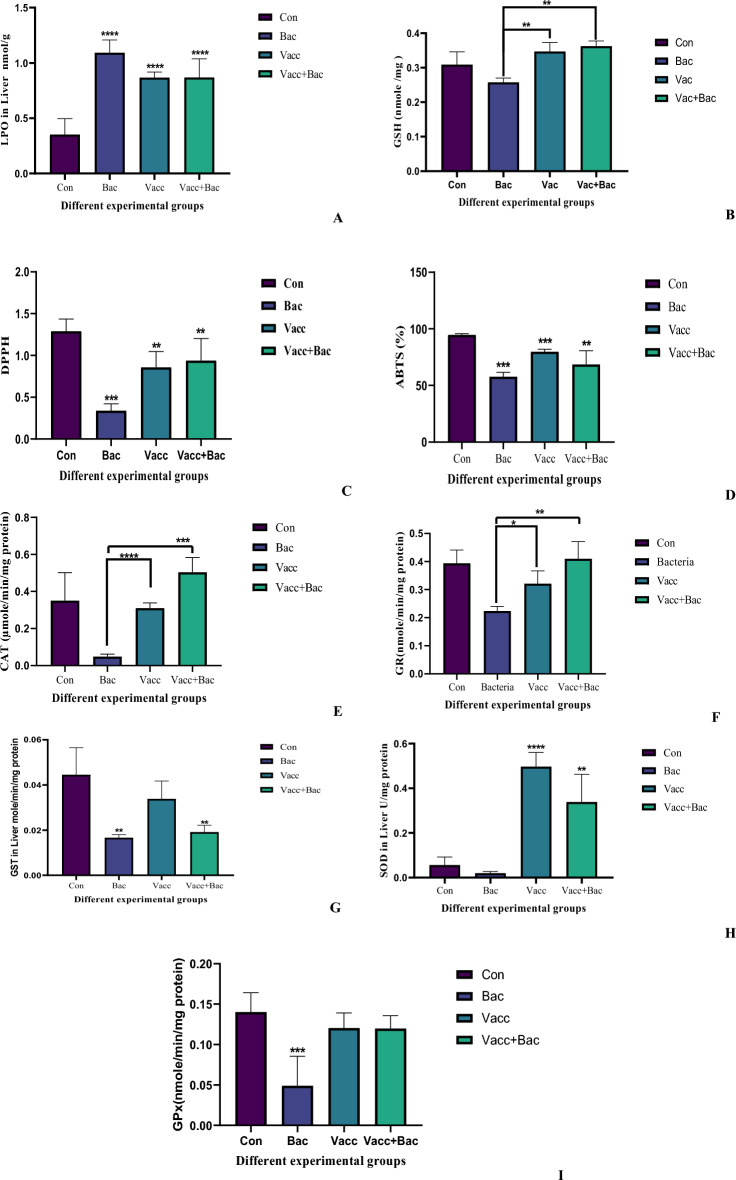
(**A**)The oxidative stress LPO, (**B**) The antioxidants biomarkers (GSH), (**C**), (DPPH), (**D**) (ABTS), (**E**) (CAT), (**F**) (GR), (**G**) (GST), (**H**) (SOD), (**I**) (GPx), Where the Con. The control (without treatment), Bac (the infected group with *S. pyogenes*), Vacc (the vaccinated group), Vacc + Bacc (Challenged group after vaccination).

The lipid peroxidation was evaluated, and the bacterial group showed a marked increase in LPO concerning the control (1.093 ± 0.1147 Vs 0.3518 ± 0.1436, *P* < 0.0001****^)^. Additionally, the Vacc and Vacc + Bac displayed marked elevation about the control (0.8680 ± 0.04964, *P* < 0.0001**** & 0.8688 ± 0.1687, *P* < 0.0001**** Vs 0.3518 ± 0.1436). In contrast, the Vacc and Vacc + Bac showed a significant decrease compared with the bacterial group. (0.8680 ± 0.04964, *P* = 0.0289*, 0.8688 ± 0.1687,* P* = 0.0296*) as shown in Fig. 2A.

The current study represents a marked decrease in bacteria total antioxidant levels using DPPH as a model in reference to the control (0.3413 ± 0.07955 Vs 1.291 ± 0.1451, *P* = 0.005***). Also, the vaccine groups and vaccine + Bacteria showed significant decreases compared with control (0.8598 ± 0.1880, *P* = 0.0010** & 0.9397 ± 0.2621, *P* = 0.0055 Vs 1.291 ± 0.1451), additionally, the vaccine and vaccine + Bacteria displayed marked increases comparing with bacteria (0.8598 ± 0.1880, *P* = 0.0172 & 0.9397 ± 0.2621,* P* = 0.0238 Vs 0.3413 ± 0.07955) as shown in Fig. 2C.

The Total antioxidants using ABTS were increased in the current study The Bacterial group represented marked decreases compared with control (57.82 ± 3.702 Vs 94.80 ± 1.018, *P* < 0.0001****), also the vaccine and Vacc + Bac showed a marked decrease in reference to control (79.91 ± 2.030, *P* < 0.0001**** & 68.69 ± 12.00, *P* = 0.0081** Vs 94.80 ± 1.018). Additionally, the Vacc + Bac showed a nonsignificant increase compared with the bacterial group (68.69 ± 12.00 Vs 57.82 ± 3.702, *P* = 0.1099) in contrast the Vacc showed a marked increase compared with the bacterial group (79.91 ± 2.030 Vs 57.82 ± 3.702, *P* < 0.0001****) as shown in Fig. 2D.

The GSH levels were evaluated as the bacterial represent a non-marked decrease (0.2575 ± 0.01253 Vs 0.3092 ± 0.03709, *P* = 0.0871), where the Vacc and Vacc + Bac showed a non-marked increase (0.3473 ± 0.02537, *P* = 0.0028**, 0.3625 ± 0.01484, *P* = 0.0007*** Vs 0.2575 ± 0.01253). In contrast, the level of GSH showed marked elevations in Vacc and VACC + Bac compared with a bacterial group (0.3473 ± 0.02537, *P* = 0.0028** & 0.3625 ± 0.01484, *P* = 0.0007***) (Fig. 2B). The Catalase results showed a significant increase compared with the control (0.04850 ± 0.01340 Vs 0.3500 ± 0.1507, *P* = 0.0186), in contrast to vaccine and vaccine + Bacteria represent non-marked decrease and increase respectively compared to control (0.3103 ± 0.02769, *P* = 0.9356 & 0.5037 ± 0.07914, *P* = 0.2106 Vs 0.3500 ± 0.1507) as in Fig. 2E. The level of liver GR represents a marked change between different groups where the bacterial groups represent a marked decrease compared with the control (0.2238 ± 0.01624 Vs 0.3935 ± 0.04742, *P* = 0.005). In contrast to vaccine and vaccine Bacteria groups which showed a non-marked decrease and increase in reference to control (0.321 ± 0.04514, *P* = 0.2132 & 0.4098 ± 0.06079, *P* = 0.9433 Vs 0.3935 ± 0.04742) as in Fig. 2F.

The GST in the liver showed significant change between all groups as the bacterial groups displayed a marked decrease compared to the control (0.01667 ± 0.001366 Vs 0.0445 ± 0.01200, *P* = 0.0097**), in contrast to the Vacc group which showed non-significantly decreased comparing with control (0.0338 ± 0.00793 Vs 0.0445 ± 0.01200, *P* = 0.1016), Additionally, the Vacc + Bac showed significantly decreased compared with control (0.01917 ± 0.002944 Vs 0.04450 ± 0.01200, *P* = 0.0048**). Furthermore, the Vacc group showed a significant increase compared with the bacterial group (0.03383 ± 0.007935 Vs 0.01667 ± 0.001366, *P* = 0.0105*). While the Bacteria + Vacc showed a non-significant increase compared with bacterial groups (0.01917 ± 0.002994 Vs 0.01667 ± 0.001366, *P* = 0.2909) as in Fig. 2G. The SOD activity in the bacterial group showed a non-significant decrease compared with the control (0.02000 ± 0.007014 Vs 0.05633 ± 0.03576, *P* = 0.2704) whereas the Vacc group showed a marked increase compared with the control (0.4967 ± 0.06387 Vs 0.05633 ± 0.03576, *P* < 0.0001****), also the Vacc + Bac showed significant increases compared with control. Additionally, the vaccine group showed a significant increase compared with the bacterial group (0.4967 ± 0.06387 Vs 0.02000 ± 0.007014,* P* < 0.0001****), Furthermore, the Vacc + Bac showed a significant increase compared with the bacterial group (0.3388 ± 0.1240 *P* = 0.0058**) as in Fig. 2H. The Levels of GPx evaluated for all groups marked decrease in the bacterial group compared with the control (0.04917 ± 0.02378 Vs 0.1403 ± 0.02378, *P* = 0.0002***). In contrast Vaccine and vaccine, + Bac showed a non-significant decrease compared with the control. (0.1205 ± 0.01865 *P* = 0.5514 & 0.1198 ± 0.01592,* P* = 0.3289 Vs 0.1403 ± 0.02378), in contrast, the Vacc and Vacc + Bac shows Significant increase compared with bacterial group (0.1205 ± 0.01865,* P* = 0.0491 & 0.1198 ± 0.01592, *P* = 0.0159, Vs 0.1403 ± 0.02378)) as in Fig. 2I.

### The anti-inflammatory biomarkers

**Figure 3:**
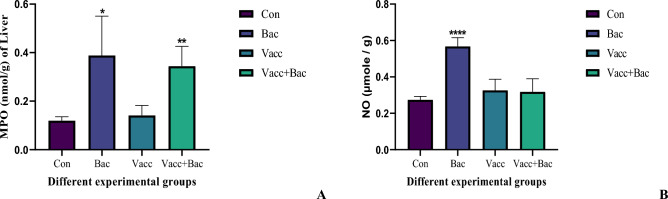
The anti-inflammatory biomarkers (**A**) (MPO) and (**B**) (NO), Where the Con. The control (without treatment), Bac (the infected group with *S. pyogenes*), Vacc (the vaccinated group), Vacc + Bacc (Challenged group after vaccination).

The NO inflammatory biomarkers represented a marked increase in bacterial groups in reference to the control (0.2745 ± 0.01853 Vs 0.2745 ± 0.01853, *P* < 0.0001****). In contrast, the levels of NO in Vacc and Vacc + Bac groups showed nonsignificant increases (0.3267 ± 0.06026, *P* = 0.1001 & 0.3185 ± 0.07123, *P* = 0.3267 Vs 0.2745 ± 0.01853), Additionally the Vacc and Vacc + Bac significant increased compared with bacterial groups (0.3267 ± 0.06026, *P* = 0.0002 & 0.3185 ± 0.07123, *P* = 00,008 Vs 0.5680 ± 0.04759) (Fig. 3B). The MPO in liver results showed a marked increase compared with the control (0.3880 ± 0.1620, Vs 0.1197 ± 0.01611, *P* = 0.0433), in contrast to the vaccine which showed a nonsignificant increase compared with the control group (0.1413 ± 0.04102 Vs 0.1197 ± 0.01611, *P* = 0.5563)., also the vaccine + Bacteria were significantly increased compared with control (0.3443 ± 0.8158 Vs 0.1197 ± 0.01611, *P* = *0.*0029) (Fig. 3A).

### Vitamin C and DNA fragmentation profile

**Figure 4:**
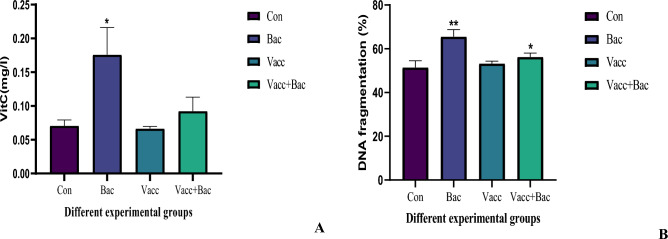
Showed Vit C (**A**) and DNA fragmentation (**B**) Where the Con. The control (without treatment), Bac (the infected group with *S. pyogenes*), Vacc (the vaccinated group), Vacc + Bacc (Challenged group after vaccination).

The DNA fragmentation was investigated in the different groups as shown in Fig. 4B. The bacterial group represented a marked increase about the control (65.57 ± 3.164 Vs 51.44 ± 3.109, *P* = 00.0016**^.^), in contrast, the Vacc group showed a non-significant increase compared to the control (53.27. ± 1.058 Vs 51.44 ± 3.109, *P* = 0.3203), additionally, the Vacc + bac group showed significantly increased compared to control (56.28 ± 1.784 Vs 51.44 ± 3.109, *P* = 0.0231), Furthermore, the Vacc showed significantly decreased in DNA fragmentation compared to bacterial group (53.27 ± 1.058 Vs 65.57 ± 3.164, *P* = 0.0018), also the Vacc + Bac showed significantly decreased compared to Bacterial group (56.28 ± 1.784 Vs 65.57 ± 3.164, *P* = 0.0020) (Fig. 4B). The VitC levels marked an increase in a bacterial group about the control (0.1760 ± 0.04017 Vs 0.07083 ± 0.008353, *P* = 0.0101*) In contrast the Vacc showed a nonsignificant decrease compared to the Control (0.06650 ± 0.003271 Vs 0.07083 ± 0.008353, *P* = 0.3780) additionally the Vacc + Bac group showed nonsignificant increased compared to control (0.09233 ± 0.02073 Vs 0.07083 ± 0.008353, *P* = 0.1648). Furthermore, the Vacc group showed a significant decrease in the bacterial group (0.06650 ± 0.003271 Vs 0.1760 ± 0.04017, *P* = 0.0049) also the Vacc + BAC showed a significant decrease compared with the bacterial group (0.09233 ± 0.02073 Vs 0.1760 ± 0.04017, *P* = 0.0387) (Fig. 4A).

### Hematological parameters indices

**Figure 5:**
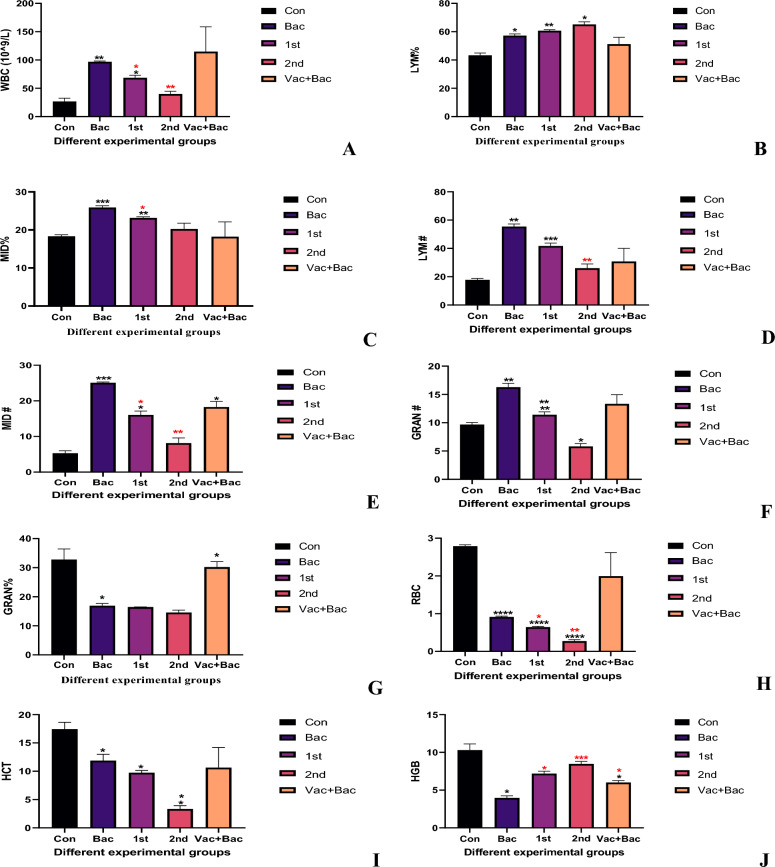

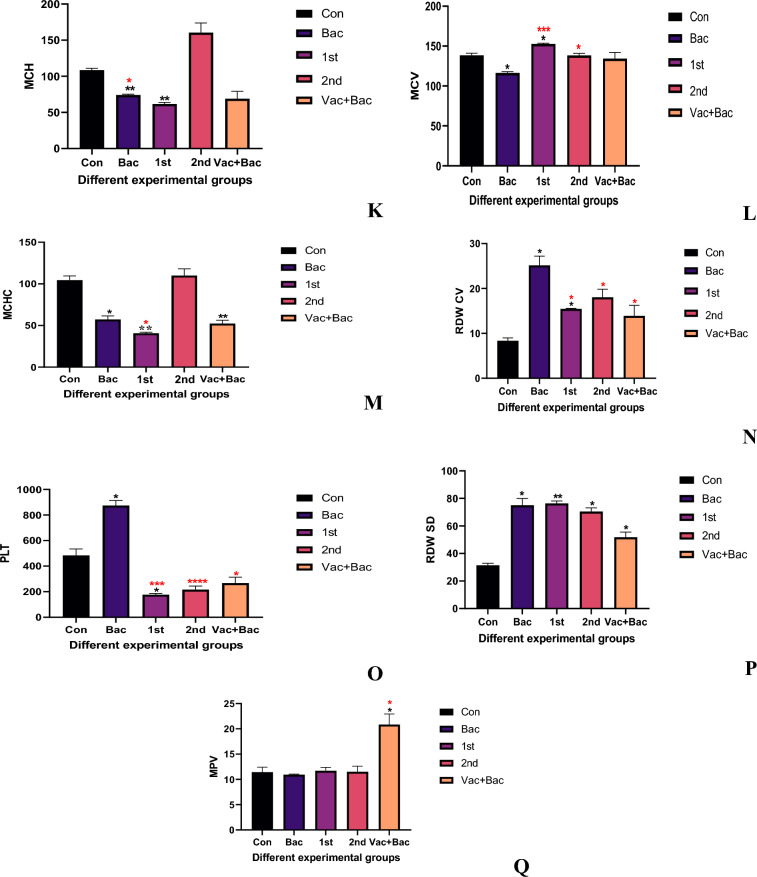
The different Hematological parameters indices in four experimental groups, Where the Con. The control (without treatment), Bac (the infected group with *S. pyogenes*), Vacc (the vaccinated group), Vacc + Bacc (Challenged group after vaccination).

The WBC showed significant changes in the bacterial group compared with the control (96.77 ± 1.514 Vs 26.70 ± 5.866, *P* = 0.0080**), also the 1st injection of the vaccine group showed a significant increase compared to the control (68.53 ± 4.332 Vs 26.70 ± 5.866, *P* = 0.049, while the 2nd and Vacc + Bac showed non-significant increasing comparing with control (39.97 ± 4.801, *P* = 0.1619 & 115.0 ± 43.66, *P* = 0.2516 Vs 26.70 ± 5.866). Additionally, the 2nd group (injection at 32) showed a significant decrease compared with (vaccine + Bacteria) (39.97 ± 4.80, *P* = 0.014* Vs 114.97 ± 43.66 *P* = 0.014*). Additionally, the vaccine without bacteria groups at 21 (1st), and at 32 days (2nd) showed a significant decrease compared with a bacterial group (68.53 ± 4.332, *P* = 0.0390* & 39.97 ± 4.801, *P* = 0.0032**). The Vacc + BAC showed a non-significant increase compared with the bacterial group (115.0 ± 43.66 Vs 96.77 ± 1.514 *P* = 0.9373) (Fig. 5A). The LYM% in the bacterial group showed a significant increase compared with the control (57.20 ± 1.217 Vs 43.30 ± 1.682, *P* = 0.0101*), furthers more the 1st and 2nd injections of the vaccine showed a significant increase compared with the control (60.70 ± 0.7211, *P* = 0.0021** & 65.17 ± 1.790, *P* = 0.0131* Vs 43.30 ± 1.682). The 1st and 2nd showed a non-significant increase while the Vacc + Bac showed a non-significant decrease compared with bacterial groups (Fig. 5B).

MID% also showed a significant change as the bacterial group showed a significant increase compared with the control (25.90 ± 0.4583 Vs 18.33 ± 0.3786, *P* = 0.0007***). Additionally, the 1st injection of the vaccine group showed a significant increase compared with the control (23.17 ± 0.2887 Vs 18.33 ± 0.3786, *P* = 0.0067**) (Fig. 5C). The 2nd injection of the vaccine showed a non-significant increase compared with the control and Vacc + Bac showed a non-significant decrease. The 1st injection was significantly decreased compared with bacterial groups (23.17 + 0.2887 Vs 25.90 ± 0.4583, *P* = 0.0063**).

GRAN% showed significantly decreased in a bacterial group compared to control (16.90 ± 0.8185 Vs 32.77 ± 3.686, *P* = 0.0422*) while the 1st, 2nd and Vacc + Bac showed non-significant decreased compared with control (16.47 ± 0.05774, *P* = 0.0509 & 14.57 ± 0.8083, *P* = 0.0579 & 30.20 ± 1.873, *P* = 0.8779 Vs 32.77 ± 3.686) (Fig. 5G). Additionally, 1st and 2nd injections showed nonsignificant decreases compared with bacterial groups (16.47 ± 0.05774, *P* = 0.8917 & 14.57 ± 0.8083, *P* = 0.2370 Vs 16.90 ± 0.8185). In contrast, the Vacc + BAC showed a significant increase compared with bacterial groups (30.20 ± 1.873 Vs 16.90 ± 0.8185, *P* = 0.0407*). Furthermore Vacc + Bac showed significant increased compared with 1st injection (30.20 ± 1.873 Vs 16.47 ± 0.05774, *P* = 0.0178*), and significant.

The MCV showed a significant increase compared with the control (116.4 ± 1.464 Vs 138.4 ± 2.610, *P* = 2.610*), also the 1st injection vaccine group showed a significant increase compared with the control (152.7 ± 0.9504 Vs 138.4 ± 2.610, *P* = 0.0441*). The 2 and Vacc + Bac showed non-significant decreases compared with control (138.2 ± 2.706, *P* > 0.9999 & 134.3 ± 7.877, *P* = 0.9359) (Fig. 5L). The 1st and 2nd injection groups showed a significant increase compared with bacterial groups (152.7 ± 0.9504,* P* < 0.0001**** & 138.2 ± 2.706*, P* = 0.0334* Vs 116.4 ± 1.464). While the Vacc + Bac group showed a non-significant increase compared with bacterial groups (134.3 ± 7.877 Vs 116.4 ± 1.464, *P* = 0.2052). The MCH bacterial group showed a significant decrease compared to the control (74.03 ± 1.270 Vs 108.6 ± 2.452, *P* = 0.0072**), also the 1st group showed a significant decrease compared with the control (61.57 ± 2.194 Vs 108.6 ± 2.452, *P* = 0.0097**), the 2nd injection group showed nonsignificant increased compared to control (160.4 ± 13.35 Vs 108.6 ± 2.452, *P* = 0.0686), also the Vacc + Bac showed non-significant decreased (68.87 ± 10.35 Vs 108.6 ± 2.452, *P* = 0.0638), the 1st injection showed non-significant decreased comparing with bacteria (61.57 ± 2.194 Vs 74.03 ± 1.270,* P* = 0.0581), while the 2nd injection showed significantly increased compared with a bacterial group (160.4 ± 13.35 Vs 74.03 ± 1.270, *P* = 0.0198*), furthermore the Vacc + Bac showed non-markedly decreased comparing with a bacterial group (68.87 ± 10.35 Vs 74.03 ± 1.270, *P* = 0.8478) (Fig. 5K).

The MCHC showed significantly decreased compared with control (57.20 ± 4.386 Vs 104.5 ± 5.085, *P* = 0.0331*), the 1st injection group showed significantly decreased comparing with control (40.70 ± 1.212 Vs 104.5 ± 5.085, *P* = 0.0097**) while the 2nd group showed nonsignificant increased comparing with control (109.9 ± 8.190 Vs 104.5 ± 5.085, *P* = 0.8131), the Vac + Bac showed significant decreased comparing with control (52.33 ± 3.950 Vs 104.5 ± 5.085, *P* = 0.0013**). The 1st injection group showed significant decreased comparing with bacterial group (40.70 ± 1.212 Vs 57.20 ± 4.386, *P* = 0.0374*), while the 2nd group showed nonsignificant increased comparing with bacterial group (109.9 ± 8.190 Vs 57.20 ± 4.386, *P* = 0.0528), the Vacc + Bac showed nonsignificant decreased comparing with bacterial group (52.33 ± 3.950 Vs 57.20 ± 4.386, *P* = 0.7111) (Fig. 5M).

The RDW CV showed a significant increase in the bacterial group compared with the control (25.17 ± 2.050 Vs 8.367 ± 0.6110, *P* = 0.0233*), also the 1st injection group showed significantly increased compared with control (15.47 ± 0.1155 Vs 8.367 ± 0.6110, *P* = 0.0109*), while the 2nd injection showed nonsignificant increased comparing with control (18.03 ± 1.804 Vs 8.367 ± 0.6110, *P* = 0.0608), also the Vacc + Bac showed nonsignificant increased compared with control (13.90 ± 2.352 Vs 8.367 ± 0.6110, *P* = 0.2186) (Fig. 5N). Additionally the 1st and 2nd group represent marked decreased compared with a bacterial group (15.47 ± 0.1155, *P* = 0.0419*& 18.03 ± 1.804, *P* = 0.0238* Vs 25.17 ± 2.050), furthermore, the Vac + Bac showed significantly decreased compared with a bacterial group (13.90 ± 2.352 Vs 25.17 ± 2.050,* P* < 0.0001****).

The RDW SD results showed a significant increase in the bacterial group in reference to the control (75.07 ± 4.900 Vs 31.40 ± 1.510, *P* = 0.0217*), the 1st and 2nd injection vaccine groups showed marked increase compared with the control group (76.40 ± 1.732, *P* = 0.0018** & 70.47 ± 2.641, *P* = 0.0118** Vs 31.40 ± 1.510). The Vacc + Bac showed a significant increase compared with the control (51.77 ± 3.753 Vs 31.40 ± 1.510*, P* = 0.0127*). Additionally, the 1st, 2nd, and Vacc + Bac groups showed non-significant decreases or increases compared with bacterial groups (Fig. 5P).

The PLT results showed a significant increase in the bacterial group in reference to the control (873.3 ± 40.61 Vs 483.7 ± 50.58*, P* = 0.0399*), also the 1st injection showed a marked decrease in reference to the control (177.3 ± 9.238 Vs 483.7 ± 50.58, *P* = 0.0336*), the 2nd and Vacc + Bac groups showed non-markedly decreased comparing with control (216.3 ± 28.29, *P* = 0.0669 & 266.7 ± 47.26, *P* = 0.1506 Vs 483.7 ± 50.58) (Fig. 5O). The 1st and 2nd injection groups showed significant decreases compared with the bacterial group (177.3 ± 9.238, *P* = 0.0006*** & 216.3 ± 28.29, *P* < 0.0001**** Vs 873.3 ± 40.61). Additionally, the Vacc + Bac group showed a significant decrease compared with the bacterial group (266.7 ± 47.26 Vs 873.3 ± 40.61,* P* = 0.0151*).

The MPV showed a non-significant decrease in bacterial in reference to the control (10.93 ± 0.1155 Vs 11.43 ± 1.002, *P* = 0.9106), also the 1st and 2nd injection groups represent a nonsignificant increase in reference to the control (11.70 ± 0.6928, *P* = 0.9952 & 11.50 ± 1.114,* P* = 0.9999 Vs 11.43 ± 1.002). While the Vacc + Bac displayed a marked increase in reference to the control (20.83 ± 2.113 Vs 11.43 ± 1.002, *P* = 0.0229*) (Fig. 5Q). Additionally, the 1st and 2nd showed a nonsignificant increase compared with the bacterial group (11.70 ± 0.6928, *P* = 0.5526 & 11.50 ± 1.114, *P* = 0.8808 Vs 10.93 ± 0.1155) Furthermore the Vacc + Bac group represent a marked increase in reference to the bacterial group (20.83 ± 2.113 Vs 10.93 ± 0.1155, *P* = 0.0458*).

### Histopathology

**Figure 6:**
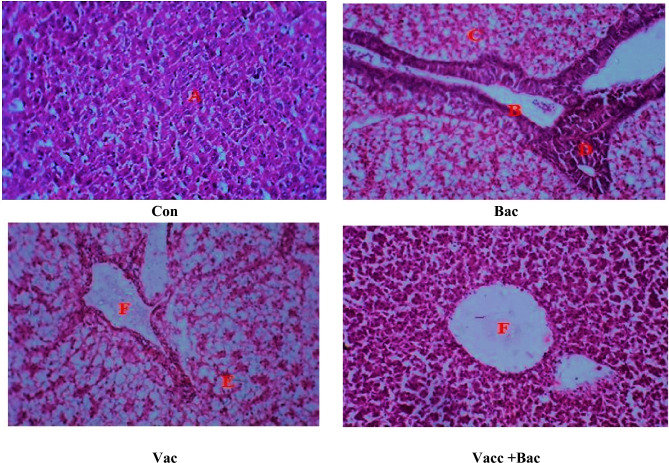
(**A**) Normal liver architecture, (**B**): Enlargement of blood vein due to inflammation, (**C**): Red lump of blood, besides focal hemorrhages and inflammation, (**D**): hemocyte aggregation in the hepatic tissues, (**E**): vacuolation, (**F**): Dilated blood vessels. Where the Con. The control (without treatment), Bac (the infected group with *S. pyogenes*), Vacc (the vaccinated group), Vacc + Bacc (Challenged group after vaccination).

Figure 6 shows the different histopathological changes in the liver of different experimental groups of *Nile tilapia* fingerlings. In the control group, there were no liver lesions and normal hepatocytes (A) were ordered and clustered in cords, separated by blood sinusoids. Additionally, polygonal hepatic cells and blood capillaries are seen to be organized normally. In the infected liver by *S. pyogenes* bacteria, severe damage, and inflammation (B) characterized by a red lump of blood (C) besides focal hemorrhages and inflammation are seen in dermal ulceration lesions. Free radicles may cause massive hemocyte aggregation (D) in the hepatic tissues because of stress and liver necrosis. The adjuvant and adjuvant + bacteria groups showing the arrangement of hepatocytes in the liver revealed vacuolation (E) and severe necrosis in some cells. Besides, lymphocytic aggregation’s focal area (F) migrates from the dilated blood vessels toward the necrotic hepatic cells.

## Discussion

The route of vaccine administration affects the quality and effectiveness of the vaccine, which is related to suitable immunization routes for stimulating the fish immune system, the intraperitoneal (IP) injection is considered the most effective route although it’s stressful to the animals and hence expensive^33^. The continuous use of different antibiotics against different types of bacterial infections raises the phenomena of bacterial resistance resulting in super bacteria^34^. One of the possible solutions for such problems is the use of a vaccine as an alternative approach to treating aquaculture fish from pathogens although the effectiveness of vaccine there are different challenges such as specificity, pathogen, inoculation, and the lack of availability of different pathogens^35^. Pathogenic bacteria cause huge losses in the global aquaculture branch *Streptococcus* is one of the most serious bacterial infections at the economic level^36^. There are different *Streptococcosis* pathogenic in fish such as *Streptococcus iniae*, *Streptococcus faecium*, *Streptococcus agalactiae*, *Streptococcus equi, Streptococcus equisimilis*, and *S. pyogenes*^37^. Few studies investigated the destructive role of *Streptococcus* concerning vaccine and antagonistic activity and in vivo efficacy of natural immunity against streptococcal agents. And importantly to know that there is no previous study on *S. pyrogen* vaccine in fish^36^.

The hematological parameters of fish and aquatic animals can be used to observe their health status and immune responses. The WBC count is necessary for both the nonspecific and specific immune systems of fish^33^. A wide range of white blood cells collaborate in the cellular response, including, monocytes, and granulocytes. The current study showed that the vaccinated fish (21 days) showed a higher number of leukocytes (WBCs) than unvaccinated fish, the first group of vaccinated fish (21 days) showed a higher number than the second group of vaccinated fish (32 days) and the third group of vaccinated fish. The WBC counts of vaccinated *Tilapia* were higher before and after the *S. pyogenes* challenge. A higher WBC count leads to more antibodies, resulting in body resistance to extraneous substances^38^. An increase in phagocytic cells is associated with an increase in WBC count in the current experiment. On the other hand, the immunized fish showed a better immune response against infection with *S. pyogenes*. In the first group, WBC, Granulocytes (GRAN %, GRAN #), and Lymphocytes (LYM % and LYM #) counts were higher in fish injected with IP, while the same counts were also higher in fish treated with IP. (Fig. 5B, D, F, G) shows that non-vaccinated fish had lower WBC, granulocyte, and lymphocyte counts. Increasing WBC was attributed to the migration of white blood cells from the spleen to the blood circulation^39^. Despite being unclear in their function, lymphocytes in fish participate in inflammatory processes. The defense mechanisms might recruit them to the focus of the lysate, which explains their high numbers in the blood circulation of infected *Tilapia*. That’s also consistent with our results which show significant increases in bacterial (infected group), but at lower levels than vaccinated groups. We hypothesized that after vaccine immunization, innate and specific immunity were activated. Many pathogenic organisms induce general and nonspecific stress and *oxidative* stress. According to several authors, bacterial challenge leads to anemia and impaired oxygen transport. Hematological parameters are a major indicator of bacterial infection in fish^40^. In the current study, HCT, HGB, and RBC significantly decreased in the bacterial group (Fig. 5H–J). By observing hematocrit, hemoglobin rate, and erythrocyte count, oxygen concentration can be related to fish health. According to Usman et al*.*^41^, with reduced erythrocyte count and hemoglobin rate, the infection impaired oxygen-carrying capacity in *Tilapia* infected by *S. iniae.* And that also seen in our study as hemoglobin was significantly decreased in fish with *S. pyogenes.* Possibly, pathogenic bacteria break down RBCs at a faster rate, and/or fewer RBCs are formed^42^. According to Hardi et al.^43^, hemoglobin content decreases due to swelling of RBCs and poor hemoglobin mobilization of the spleen. The significant reduction of hemoglobin after fish infection could be attributed to severe anemia. As a result of the destruction of intestinal cells that produce vitamin B12, the hemoglobin portion of red cells^44^, the maximum value of MCV was found in the 1st (21 days) vaccinated group. The results also showed that the injection with the vaccine with varying times markedly increased MCV, while markedly decreasing the value of MCH and MCHC after infection of the *Nile tilapia* with *S. pyogenes* and injection of vaccine at 21 days was decreased while at 32-days vaccination (2nd group) MCH and MCHC was increased. WBC counts in the *Nile tilapia* fed different concentrations of vaccine were markedly higher, compared to the control group) According to Sutili et al.^45^, Platelets are classically known as essential mediators of hemostasis and thrombosis. Recently, platelets have been recognized for their inflammatory functions that modulate the immune response. The immunoreceptors on platelets allow them to recognize intravascular pathogens as sentinels. In response to activation, platelets release antimicrobial proteins (AMPs) and activate immune cells to clear pathogens. However, aberrant platelet activation can lead to inflammation and thrombotic events^46^. Platelet count increases under physiological conditions as a result of progenitor megakaryocyte reactivity, and during infection^47^.The RDW level increase has several clinical conditions, for example, in response to ineffective red cell ].uction, which can be caused by deficiencies in iron, vitamin B12, or folate. When compared with the bacterial (infected) group, the vaccinated group exhibited higher hematocrit levels and that can explain our results as the RDW was increased markedly in the group injected with the pathogen (GroupII) Fig. 5N and P.

According to Santika et al.^48^, the elevation of HCT levels of vaccinated *Tilapia* tends to induce an increase in HCT levels to a certain degree. A high HCT indicates healthy blood and the ability to bind oxygen. Both infection and injection vaccination treatments reduce hematocrit levels compared with controls (unvaccinated and uninfected). It is caused by the infection of *S. pyogenes.* According to Anderson et al.^49^, a decrease in HCT indicates a pathogen infection in fish. Additionally, the decreased hematocrit level after the bacterial infection is also due to decreased appetite after the *S. pyogenes* infection, low hematocrit can indicate a lack of protein, vitamin deficiencies, or infection of the fish. A marked elevation in the HCT value after the challenge test suggests that the blood cells play an important role in increasing the fast response in adequate quantities to relieve pathogenic bacteria infection. *Tilapia* had marked changes in HCT values on days 21 and 32 following vaccination. However, the RBCs were lower in fish vaccinated at 32 and elevated markedly after the challenge. Nevertheless, these values were similar to those observed in *Tilapia* with no stress stimulation in Martins et al.^50^. The vaccinated fish had higher total RBCs and HGB percentages on day 21 than the bacterial group (injected with only bacteria) (Fig. 5J, H). Martins et al.^50^ identify *Tilapia* glycemia as a stress factor. Nevertheless, in this study, glucose was stable in all fish except those submitted to the challenge after vaccinated vaccination on 32 days (*P* = 0.008) Fig. 1A. Martins et al*.*^50^, Okamura et al.^51^ found similar indices among healthy *Nile tilapia*. There is evidence that high serum proteins are likely due to an improvement in fish non-specific immune responses^52,53^. In the present study, the vaccinated groups (vaccinated only and challenged vaccinated after 32 days) markedly increased in reference to other groups (control and infected group) vaccinated group documented a notable (*P* = 0.0006***) elevation in total protein level, this finding in the present study agree Viji et al*.*^54^ reported higher albumin level in biofilm groups than that of the control group Fig. 1C. The current studies evaluate the oxidative stress and antioxidant defense effects of vaccination against *S. pyogenes* in *Nile Tilapia* liver. The oxidative stress markers (Malondialdehyde), antioxidant defenses (SOD, CAT, GR, GSH, GPx, and GST) Fig. 2H, E, F,B, I and G, and the total antioxidant capacity of *Nile Tilapia* were measured (DPPH and ABTS) Fig. 2C & D).Our study showed that *Nile Tilapia* vaccinated against *S. pyogenes* showed alterations in antioxidant defenses and oxidative stress. Additionally, the results revealed that vaccination against *S. pyogenes* caused liver tissue to undergo lipid peroxidation and that was confirmed and indicated in the histology study which showed several types of tissue damage and inflammatory response in reference to control (uninfected group) Fig. 4. It is important to note that, in vaccinated groups (with or without challenge), the immune system can reestablish its pro- and antioxidant balance after vaccination. Detection of antioxidant enzymes reveals the health of the body’s antioxidant system, letting you know what the system is capable of when it comes to the digestion of oxygen-free radicals and protection of the tissue from oxidative damage. The rate of oxidative damage can be increased by many factors (disease, toxins, immunization, aging, and exercise), resulting in oxidative stress^55^. Depletion or elevation of ROS causes oxidative stress when the balance between oxidants and antioxidants is disrupted^56^. Free radicals are dangerous, but despite their potential danger, cells have various defense mechanisms against them^57^. The present study established that fish vaccinated against *S. pyogenes* have elevated levels of lipid peroxidation than fish immune systems^58^. SOD and CAT enzymes are the first line of defense against oxidative stress and are often used to measure ROS production^59^. The development of many vaccines to decrease the infection load as well as protect fish in aquaculture from acute bacterial infections such as *Vibriosis, Streptococcus*, *Edwardsiellosis, Yersiniosis*, and *Furunculosis* is ongoing^60^. After vaccination, there was a notable decrease in liver lipid peroxidation indicators observed in our study which showed a decrease in LPO in reference to the infected group with *S. pyrogen* Fig. 2A. The liver is a metabolically active and highly antioxidant organ. Consequently, compared to the other tissues, the liver of the infected group (the bacterial group) displayed greater levels of oxidative stress. The evidence from other studies agrees with our analysis^61^. The degree of oxidative damage under the influence of *S. pyogenes* is demonstrated by the markedly higher levels of oxidative stress biomarkers in the liver of the infected group of bacteria than in the fish that received vaccinations. Skugor et al*.*^62^ used multiple gene expression profiling to outline the mechanisms that determine the success of vaccine protection against pathogens like NF-kB. By upregulating NF-kB and AP-1 proteins in response to pathogens and cytokines, pathogens and cytokines induce a massive release of immune mediators and effector proteins into the body. Free radicals, and genotoxic agents, such as free radicals, are a few factors that damage cells, including NF-kB and Jun proteins, which affect cell growth, growth arrest, DNA repair, differentiation, and apoptosis. NF-kB can also activate protection against oxidative and cellular stress by providing anti-apoptotic and proliferation-promoting signals and that was in agreement with the current study which investigated the level of DNA damage in all groups where the bacterial group (infected) showed significant increases in DNA damage compared with control in contrast to vaccine group without challenge and that can be explained with the results of VitC in the current study which showed in Parallel to DNA damage results as shown in Fig. 3B, which indicted the DNA damage test showed a marked increase in the bacterial group while other group showed a non-markedly increased or decreased and that may be due to highly level of oxidative stress which lead to DNA damage as a defense mechanism the Vit C increase greatly to restore the GSH molecule which in turn restore the antioxidant enzymes to help to repair the cell damage and decreased oxidative stress^63,64^ similar study for Skugor et al*.*^62^ showed that genes for proteins involved in the regulation of redox status and protection against ROS had higher expression levels in vaccinated fish with high resistance to *furunculosis*^62^. ROS in cells may lead to an elevation of antioxidant enzymes as a defense mechanism. Providing cells with a comprehensive defense against ROS-induced damage is the primary function of antioxidants. There are several defense mechanisms included in these defenses, including low molecular weight compounds (e.g., glutathione and ascorbate), and our results indicate that GSH and Vitamin C levels are elevated in the vaccine group’s Figs. 2B and 3A^65^. Antioxidant enzymes (including SOD, GST, and GR) are also elevated. An important cofactor for several glutathione-dependent antioxidant enzymes is glutathione (GSH), a tripeptide that can neutralize ROS and act as a cofactor for glutathione (GSH). As observed in our study the GSH elevated greatly in vaccinated groups (without and with challenge) and decreased in the bacterial group (infected group) the same manner was observed in other antioxidant parameters such as GST, SOD, GR, and GPx^66^, (Fig. 2B, E–I).

Antioxidant enzymes SOD, CAT, and GPx protect against oxidative stress by converting superoxide radicals into hydrogen peroxide, then water, and molecular oxygen. An important defense against oxidative stress in fish is the induction of antioxidant enzymes. Superoxide dismutase is a group of metalloenzymes responsible for dismutating superoxide into hydrogen peroxide in aerobic organisms and playing an essential antioxidant role^67^. Compared to the control group, the vaccinated group showed significantly higher SOD activity. Following immunization, an adaptive response could be observed that neutralizes ROS and also prevents membrane lipid peroxidation, especially when combining Fe^3+^ and oxygen-generating systems. Tkachenko et al.^68^ showed that It appears that, in response to oxidative stress, the decreased CAT activities in muscles and gills of vaccinated trout indicate that their capacity to scavenge hydrogen peroxide has been reduced. The three most important antioxidant enzymes that contribute to antioxidant defenses against oxidative stress are glutathione peroxidase, reductase, and transferase. As an antioxidant, GR plays an important role both in protecting cellular membranes from free radical damage as well as regulating metabolic pathways in a cell. In an NADPH-dependent reaction, glutathione disulfide is reduced to reduced glutathione when this enzyme catalyzes the reduction process. Pei et al.^69^ reported that the liver had significantly higher GR activity than other tissues, most likely due to the availability of NADPH in the liver tissues^69^. A variety of enzymes are capable of inactivating lipid-derived hydroperoxides, including selenoperoxidases. There are two types of selenoperoxidases in cells: classical GSH-peroxidase (GPx), which acts on relatively polar substrates like H_2_O_2_ and fatty acid hydroperoxides, and phospholipid hydroperoxide GSH-peroxidase. The glutathione disulfide-dependent GR is required for GPx. Oxidative stress and cytotoxicity result from a decrease in glutathione-mediated antioxidant defenses, whereas elevated levels of intracellular GSH are regarded as adaptive responses to oxidative stress. Comparing *Tilapia* vaccinated with the bacterial group, the total antioxidant capacity in the liver was significantly higher. As a result of impairment in enzymatic and non-enzymatic antioxidant synthesis, levels of cellular total antioxidant may be reduced in the infected group^70^. In vaccinated fish, glutathione-mediated antioxidant defense as well as endogenous CAT is critical for intracellular antioxidant defense. However, the antioxidant defenses of vaccinated fishes were markedly higher, probably due to liver functional activity. In *Tilapia,* glutathione-mediated antioxidant defenses were also demonstrated to protect against endosulfan-induced oxidative stress. Analysis of oxidative stress parameters showed marked differences among fish vaccinated against *S. pyogenes*. Oxidative stress and tissue responses are strongly correlated. Infected fish (bacterial group) (Fig. 2A showed an elevated level of lipid and protein oxidation biomarkers (Fig. 1B)^62^. A vaccination increased liver antioxidant defenses and made liver tissue more susceptible to oxidative damage Fig. 4 The results suggest that vaccination against *S. pyogenes* induced oxidative stress. A vaccination can restore liver tissue’s pro- and antioxidant balance. Infection and imbalance of the immune system through infectious agents result in an elevation of inflammatory systemic response^68^, and that showed in the levels of MPO and NO results Fig. 6A and B, leading to an elevation of LPO. Consequently, inflammation induced by infectious stimuli involves the reciprocal control of major mediators (COX-2, NO, ROS, and glutathione). In agreement with our results, COX-2 converts arachidonic acid into inflammatory prostaglandins with the consequent release of cytokines in consequence leads to elevated levels of both NO and MPO which were elevated in the infected group (Fig. 6A, B), and decreased markedly in the vaccinated group. There are many mechanisms behind liver tissue injury. The main cause of liver injury is free radicals^71^. They damage biological membranes by lipid peroxidation, resulting in liver cell degeneration and necrosis, and an increase in lipid peroxides in the body. MDA is the product of lipid peroxidation. Deficiency in anti-oxidants is indirectly related to membrane peroxidation and dehydration. A continuous invasion of streptococci scavenges MDA by antioxidant enzyme systems, which are products of lipid peroxidation. Indirectly, its content reflects the body’s antioxidant capacity and levels of membrane peroxidation. Antioxidant enzymes begin to play an increasingly important role in radical scavenging as *Streptococci* continue to invade. As infection time extended, antioxidants and other activities decreased, aggravating liver damage^72^.

## Conclusion

The current study focuses on the effect of vaccine (formalin-killed bacteria) before and after infection on *Nile tilapia* immune enhancement and bacteria resistance. Considering the hematological parameters, immunological responses, and protection results in this study, we concluded that the vaccine has the effect of enhancing protective immunity against *S. pyogenes.*

## Data Availability

All data available in the manuscript.
